# Harnessing Deep Learning in Ecology: An Example Predicting Bark Beetle Outbreaks

**DOI:** 10.3389/fpls.2019.01327

**Published:** 2019-10-28

**Authors:** Werner Rammer, Rupert Seidl

**Affiliations:** Department of Forest and Soil Sciences, Institute of Silviculture, University of Natural Resources and Life Sciences (BOKU) Vienna, Vienna, Austria

**Keywords:** deep neural networks, ecological prediction, machine learning, computational ecology, forest disturbance

## Abstract

Addressing current global challenges such as biodiversity loss, global change, and increasing demands for ecosystem services requires improved ecological prediction. Recent increases in data availability, process understanding, and computing power are fostering quantitative approaches in ecology. However, flexible methodological frameworks are needed to utilize these developments towards improved ecological prediction. Deep learning is a rapidly evolving branch of machine learning, yet has received only little attention in ecology to date. It refers to the training of deep neural networks (DNNs), i.e. artificial neural networks consisting of many layers and a large number of neurons. We here provide a reproducible example (including code and data) of designing, training, and applying DNNs for ecological prediction. Using bark beetle outbreaks in conifer-dominated forests as an example, we show that DNNs are well able to predict both short-term infestation risk at the local scale and long-term outbreak dynamics at the landscape level. We furthermore highlight that DNNs have better overall performance than more conventional approaches to predicting bark beetle outbreak dynamics. We conclude that DNNs have high potential to form the backbone of a comprehensive disturbance forecasting system. More broadly, we argue for an increased utilization of the predictive power of DNNs for a wide range of ecological problems.

## Introduction

Ecology is a relatively young discipline, and many of its theoretical foundations are less than a century old (Real and Brown, 1991). In recent decades, ecology has matured considerably as a scientific field, which is *inter alia* reflected by a strong increase in the application of ecological knowledge, data, and methods (e.g., Shea and Chesson, 2002), as well as a recent push towards predictive ecology (Clark et al., 2001; Evans et al., 2012; Dietze et al., 2018). Ecological prediction broadly describes the process of putting ecological knowledge, data, and methods to use for making testable, quantitative estimates about future states of an ecosystem (Luo et al., 2011). The increasing focus on prediction is motivated, amongst other things, by the growing realization that ecology is central to addressing a number of the most pressing challenges faced by humanity in the 21^st^ century, such as to mitigate the impacts of climate change and halt biodiversity loss (Mouquet et al., 2015). Providing essential ecosystem services to society while retaining the earth within its planetary boundaries (Steffen et al., 2015) requires accurate and timely forecasts of ecosystem trajectories. Consequently, policy makers and ecosystem managers look to scientists for providing the predictions needed to anticipate and manage global change (Clark et al., 2001).

Achieving precise and unbiased ecological predictions is more feasible today than ever before. This is the result of three simultaneous developments: First, the availability of ecological data has increased dramatically. With the advent of big data in ecology the field is in a rapid transition from an era characterized by data limitation, to one that is dominated by a wealth of data (Peters et al., 2014). Contributing to increasing data availability is the proliferation of remote sensing (Kennedy et al., 2014), large international research networks such as NEON and Fluxnet (Ershadi et al., 2014), and the use of citizen science (Jordan et al., 2015). Furthermore, the field has experienced a fundamental change in research culture in recent years, towards making ecological data accessible to the public (Whitlock, 2011). Second, recent methodological advances in the field of ecological modeling, data analysis, and statistics have drastically increased our ability to interface the growing amounts of data with our understanding of ecological systems. Given that we are facing a future characterized by no analog conditions (Williams and Jackson, 2007), such improvements in process understanding are a crucial prerequisite for successful ecological prediction (Evans, 2012). Finally, increasing computational power in general and a growing availability of high performance infrastructure for scientific computing in particular provide the technological backbone supporting both previously outlined trends. All three of these recent developments are important factors behind the recent proliferation of machine learning in ecology.

Machine learning (ML) is a family of computational algorithms that is concerned with identifying structure in complex, often nonlinear data, and generating accurate predictive models based on such data (Olden et al., 2008). Compared to classical statistical approaches such as regression, machine learning focuses on the use of computation to determine and describe complex relationships, and emphasizes predictive power over estimating parameters and confidence intervals (Breiman, 2001; Goodfellow et al., 2016). Situated at the intersection of computer sciences and statistics, and forming the core of artificial intelligence and data science, ML is a rapidly growing field (Jordan and Mitchell, 2015).

Deep learning is a relatively recent development in ML. Its main tool, the deep neural network (DNN), builds upon Artificial Neural Networks (ANNs) which were already conceived in the middle of the last century. Essentially, “deep learning” refers to a set of techniques that allow the training of larger (more neurons) and deeper (more layers) ANNs (Nielsen, 2015). These high capacity networks became possible due to the development of improved algorithms for optimizing connection weights [e.g., stochastic gradient descent (Rumelhart et al., 1986)] and a steep increase in available computing power and training data (Goodfellow et al., 2016). While these improvements may seem only gradual, current DNNs not only outperform their simpler ANN ancestors, but frequently also perform better than other ML approaches in standardized tests of prediction accuracy (e.g., Krizhevsky et al., 2012; Johnson et al., 2016; Szegedy et al., 2016).

In ecology, ML approaches were still rarely used a decade ago (Olden et al., 2008), but have seen a tremendous increase in popularity in recent years (Jordan and Mitchell, 2015). Yet, their potential is far from fully exploited, and deep learning applications in ecology remain scarce to date (see **Supplementary Material S1** for a full literature review). The overall aim of this work is to contribute to a wider recognition of deep learning in ecology (see also Reichstein et al., 2019) by demonstrating its potential for prediction based on an example application for which all necessary data and code are made available for the community. Specifically, we here chose bark beetle outbreaks in conifer-dominated forests as our example.

Bark beetles are important disturbance agents in forests around the world (Raffa et al., 2008). As a result of climate change bark beetle activity is expected to increase in the future (Seidl et al., 2017). The profound change in the structure and function of forests resulting from bark beetle outbreaks can have negative impacts on the provisioning of ecosystems services (such as clean water, timber, and climate regulation) to society (Thom and Seidl, 2016). However, given a timely knowledge of outbreak hotspots managers can contain beetle spread via removing susceptible trees and employing pheromone traps, making prediction of beetle risk a crucial task in forest management (Hlásny et al., 2019). However, these management measures are frequently applied based on ad hoc decisions of managers in the field, rather than on data-driven approaches quantifying bark beetle infestation probability.

Here we show how to predict bark beetle outbreak dynamics based on widely available information sources using deep learning. Specifically, we use a DNN (1) to estimate bark beetle outbreak probabilities based on outbreak patterns from the recent past, and (2) to predict the temporal dynamics of bark beetle outbreak waves.

## Materials and Methods

### Artificial Neural Networks and Deep Learning

**Figure 1:**
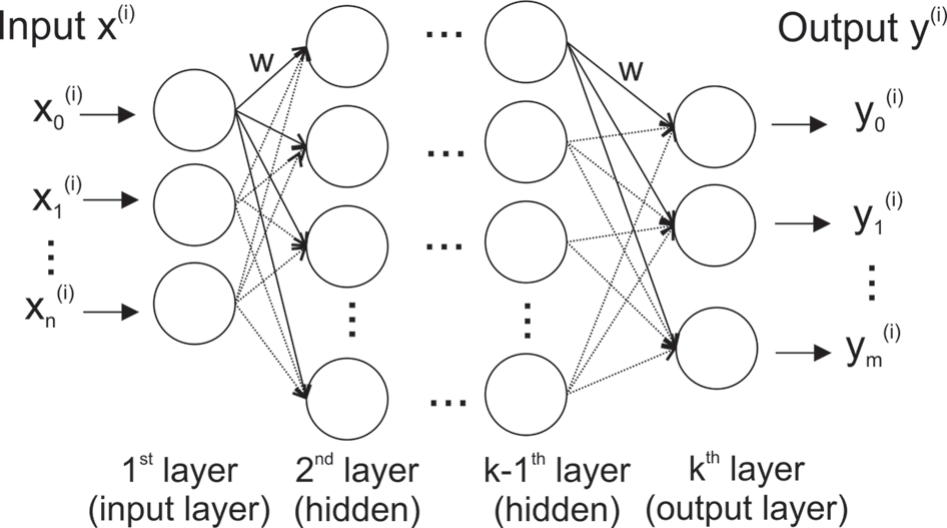
Stylized structure of a deep feedforward neural network. Each of the *k* layers consists of a variable number of fully connected neurons (circles). Thenetwork has as many neurons in the input layer as input variables (*n*), and – for classification – as many output neurons as there are classes in the data (*m*). A neuron is connected to all neurons in the two adjacent layers via a weighted connection (*w*).

The basic structure of an artificial neural network (ANN) and a deep neural network (DNN) is loosely modeled after the way biological nervous systems process information (**Figure 1**). The network consists of interconnected compute units (neurons) that are organized in layers — typically an input layer (with the number of neurons corresponding to the number of input variables), hidden layer(s), and an output layer (where each dependent variable corresponds to a neuron).

The most widely used DNN type is a feedforward neural network (often also called perceptron) (Goodfellow et al., 2016). In such a feedforward network, each neuron is connected to all neurons in adjacent layers, but neurons within a layer are not connected (**Figure 1**). The connection strength between two neurons is given by an adjustable connection weight *w*. Every neuron calculates a single output value by computing a weighted sum of the inputs and then passing the result through a non-linear function.

An extension of a feedforward network frequently used for pattern recognition in images or time series data is the convolutional neural network (CNN). In addition to fully connected layers a CNN includes multiple stages of so-called filtering and pooling layers (LeCun et al., 2015). Filters are applied locally (e.g., to detect an edge within a subset of pixels), but use the same learned weights for all elements of the data (e.g. all the pixels in an entire image). Subsequent pooling layers merge the filter outputs into more abstract representations that are less sensitive to small shifts and distortions in the data. A series of such filtering and pooling layers is able to extract increasingly abstract patterns in data.

In the training phase of a DNN the connection weights (*w* in **Figure 1**) between neurons are iteratively updated by a training algorithm to minimize the prediction error over the training data set (see **Supplementary Material S2** for more details). In order to gauge the accuracy of predictions for new input data (i.e., data not used during training), the available data is frequently split into a training data set (used for training), and a test data set. The details of the network architecture, such as the size of the network, the selection of specific layer types, and parameters of the training process strongly determine the prediction accuracy of the network and are usually problem specific. More information on deep learning is available in e.g., Angermueller et al. ( 2016), LeCun et al. (2015) and Nielsen (2015). Moreover, the **Supplementary Material S2** provides a guide for designing and training of DNNs, as well as practical considerations for DNN applications.

### A Deep Neural Network to Predict Bark Beetle Outbreaks

The following section presents an application of deep learning in the context of forest ecology, specifically predicting attacks by bark beetles. The example demonstrates the steps required for using DNNs, i.e. data preparation, network design and training, and finally making predictions using the trained DNN. All code and data required for reproducing the example presented here are available online https://github.com/werner-rammer/BBPredNet.

We used a 23-year time series of bark beetle (*Ips typographus* L.) infestation at the Bavarian Forest National Park (Germany), determined from annual aerial surveys at 30 m horizontal resolution over an area of 13,319 ha. The complete data set is available online (Seidl et al., 2015). More information on the dataset as well as statistical analyses of the data are provided by Seidl et al. (2016).

Our goal was to predict the risk of beetle attack for each 30 m cell and year, based on climatic information and the local outbreak pattern in the preceding two years. We used a DNN to predict the probability of bark beetle attack based on the following variables: the potential host cells in the vicinity of the target cell (i.e., grid cells in the local neighborhood (19 × 19 cells) that contain mature trees of the host tree species, Norway spruce (*Picea abies* (L.) Karst.), representing host connectivity in the vicinity of a target cell), the observed cells attacked in the last two years in the vicinity of a cell (representing local bark beetle pressure), average climatic conditions (i.e., the long-term mean annual temperature for the target cell, representing the variable climatic viability for bark beetle development at the landscape scale), and a qualitative classification of the outbreak stage at the regional scale (representing the surrounding forest area of Austria, Czech Republic, and Bavaria) in the previous two years (three classes, “background”, “culmination”, “gradation”, determined by using the 33^rd^ and 66^th^ percentile of the annual timber disturbed as cutoffs), representing a broad classification of population dynamic as frequently used by forest managers). We thus used both time-invariant and temporally variable predictors in our modeling, and deliberately restricted the predictors to information that was previously published (Seidl et al., 2016) and is readily available at large spatial scales for forest managers and national park services.

The full data set comprised of 1.87 million data points for training the DNN. Each training example *x_i_* consisted of the cells in a moving window around the focal cell (window size of 19 × 19), describing the local host tree distribution and the outbreak activity in the preceding two years, as well as the two auxiliary variables mean annual temperature and outbreak stage. Each *x_i_* thus contained 19 x 19 = 361 + 2 = 363 input variables. The size of 19 × 19 cells represents a rectangular area with a distance from the target cell of between 270 and 382 m in each direction, which corresponds to the dispersal distance of >95% of the bark beetles (Kautz et al., 2011). The response variable *y_i_* was the state of the focal cell (disturbed/undisturbed) in the current year.

### Experiments

#### Experiment 1: Predicting Disturbance Probability for Individual Years

In this experiment we were interested in the capability of the DNN to predict infestation probabilities for individual years. This setup resembles an application scenario where one tries to predict future disturbance from the observed disturbance pattern of the recent past. In order to include a wide range of conditions in the test dataset, we randomly selected one year from each of the three outbreak stages (background: 1993, culmination: 1997, and gradation: 2005) as test set, while all the other years were used for training the DNN. The training set for this experiment consisted of 1.58 million data points, and the test set of 292,559 cases (15.7% of all data points).

#### Experiment 2: Predicting Disturbance Dynamics Over Time

The goal of the second experiment was to test the ability of the DNN to model the temporal disturbance dynamics observed for the Bavarian Forest National Park. We randomly selected 373,817 data points (20%) from the full dataset and set them aside as test set. The remaining 80% were used for training the DNN. This experiment does not reflect forward prediction, but rather aims at scrutinizing the ability of the DNN to capture the pulse dynamics of bark beetle outbreaks.

### Model Design and Evaluation

**Figure 2:**
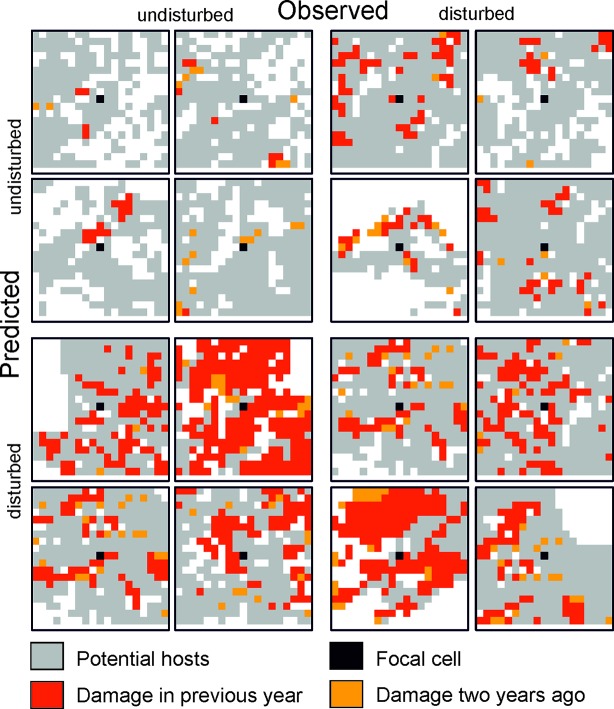
Selected examples for 19 × 19 cell matrices (grain: 30 m) from the test dataset for which the state of the focal cell was predicted correctly (top left and bottom right quartet) and incorrectly (top right and bottom left quartet).

Predicting bark beetle disturbance from infestation maps (see **Figure 2** for examples) can be viewed as a specific case of an image classification problem, where the network is asked to classify the focal cell of an example image either as disturbed or undisturbed. Neural Networks using convolutional layers (CNNs) are frequently applied for image classification (e.g., Krizhevsky et al., 2012; Szegedy et al., 2016), and were thus selected as the network type here. We used the dataset of Experiment 1 (setting aside individual years) for evaluating different network architectures. The hyper-parameters evaluated iteratively were network capacity (number of layers and neurons per layer), applied regularization techniques, as well as the used loss function and optimizer. The training of the individual candidate networks was stopped when the accuracy of the network on the test dataset did not increase further. The thus determined network architecture was also used for Experiment 2 (Abadi et al., 2016). All experiments and predictions were conducted using the TensorFlow framework and run on a desktop PC with an Intel QuadCore CPU (Intel i5-6600) and equipped with an NVidia GTX 1070 GPU.

We evaluated the network performance by calculating a number of different performance measures based on comparing predictions to test set data. The accuracy (defined as the ratio of correct classifications relative to the total number of examples) has only limited value as an evaluation metric here, as the class distribution is very unbalanced (only 3.48% of the data points are classified as disturbed). We therefore also calculated precision, recall, and the F1 Score, as well as Conditional Kappa and the True Skill Statistic (Allouche et al., 2006; Powers, 2011) (**Table 1**). Conditional Kappa and True Skill Statistic range from −1 to +1, where +1 indicates perfect agreement with test data, and values >0 indicate a performance than is better than random draws. The output of the network was a continuous disturbance probability, which was converted to a binary classification by selecting the threshold probability that yielded the highest F1 score for the training dataset. For Experiment 2 we also calculated Gleichlaeufigkeit (Buras and Wilmking, 2015), which is a measure for the similarity of two time series based on the sign of the difference between two consecutive years. Subsequently, we compared the Gleichlaeufigkeit of the DNN with the value achieved by a statistical model presented for the same system (Seidl et al., 2016).

**Table 1:** Measures for evaluating the performance of the DNN. *N* = number of examples, *tp, tn, fp; fn*, values of the confusion matrix; *tp, true positive, tn, true negative, fp, false positive, fn, false negative*.

Measure	Equation
Accuracy	$\frac{\mathrm{tn}+\mathrm{tp}}{N}$
Precision	$\frac{\mathrm{tp}}{\mathrm{tp}+\mathrm{fp}}$
Recall	$\frac{\mathrm{tp}}{\mathrm{tp}+\mathrm{fn}}$
F1 Score	$\frac{2\cdot \mathrm{precision}\cdot \mathrm{recall}}{\mathrm{precision}+\mathrm{recall}}$
Conditional Kappa	$\frac{\mathrm{precision}-\frac{\mathrm{tp}+\mathrm{fn}}{N}}{1-\frac{\mathrm{tp}+\mathrm{fn}}{N}}$
True Skill Statistic	$\mathrm{precision}+\frac{\mathrm{tn}}{\mathrm{tn}+\mathrm{fn}}-1$
Gleichlaeufigkeit	$G_{\mathrm{ix}}=\left\{\begin{array}{c} x_{i+1}-x_{i}>0:+\frac{1}{2}\\ x_{i+1}-x_{i}=0:0\\ x_{i+1}-x_{i}>0:-\frac{1}{2} \end{array}\right\}$ $G_{\mathrm{xy}}=\frac{1}{n-1}\sum_{i=1}^{n-1}\mathrm{abs}(G_{\mathrm{ix}}+G_{\mathrm{iy}})$

To better contextualize the performance of the DNN, we repeated Experiments 1 and 2 using other widely used classification algorithms, i.e., distributed random forest, gradient boosting machine, and generalized linear model. We used the H2O platform (H2O.ai, 2017), which provides a set of fast and scalable learning algorithms and is integrated within the R software and environment.

## Results

### A DNN for Predicting Bark Beetle Outbreaks

We used a convolutional neural network with five convolution layers, followed by five fully connected layers and a final softmax layer (Nielsen, 2015) for classification (for details and terminology see **Supplementary Material S2**). We used categorical cross entropy as cost function, and weight decay (Nielsen, 2015), dropout (Srivastava et al., 2014), and batch normalization (Ioffe and Szegedy, 2015) to improve generalization. **Figure S2** in the **Supplementary Material** shows the schematic structure of the DNN architecture. The presented network efficiently combines image-like pixel data with additional variables that are both numerical (climate variables) and categorical (outbreak stages). We trained the final architecture for 60 epochs, which took approximately one hour on the hardware used, and selected the epoch with the highest test set accuracy for prediction. The GitHub repository (https://github.com/werner-rammer/BBPredNet) contains the full source code for reproducing this example, and includes further details on data preprocessing and the final network architecture.

### Predictions

#### Experiment 1: Predicting Disturbance Probability for Individual Years

**Figure 3:**
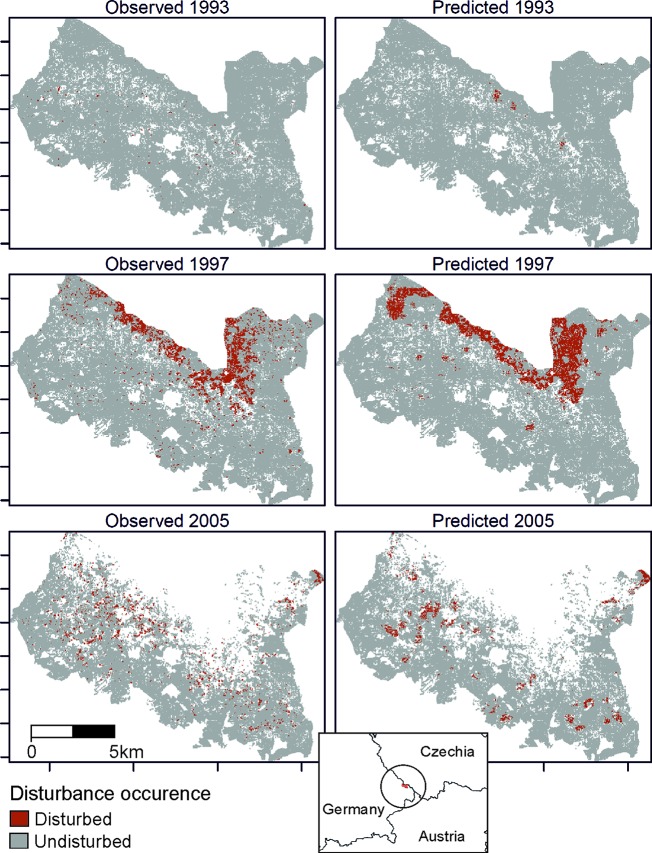
Observed (left) and predicted (right) bark beetle disturbance in the Bavarian Forest National Park for the years 1993 (background stage), 1997 (gradation stage), and 2005 (culmination stage).

The trained DNN was well able to predict the general disturbance level and spatial pattern observed in the years 1993, 1997, and 2005, i.e. in background, culmination, and gradation stage of the outbreak, respectively (**Figure 3**). The achieved accuracy was 0.966, with a precision of 0.652 and a recall of 0.392 (**Table 2**). The achieved conditional kappa and true skill statistic, which are less sensitive to the uneven class distribution inherent to disturbance data, were 0.637 and 0.626, respectively. **Figure 2** shows selected examples for both successful and unsuccessful classifications taken from within the landscape, illustrating the cell-level stochasticity of bark beetle activity.

**Table 2:** Performance measures for the two experiments. See Table 1 for details.

Parameter	Experiment 1 (n = 292,559)	Experiment 2 (n = 373,817)
Accuracy	0.966	0.959
Precision	0.652	0.413
Recall	0.392	0.411
F1 Score	0.490	0.412
Conditional Kappa	0.637	0.392
True Skill Statistic	0.626	0.392

#### Experiment 2: Predicting Disturbance Dynamics Over Time

**Figure 4:**
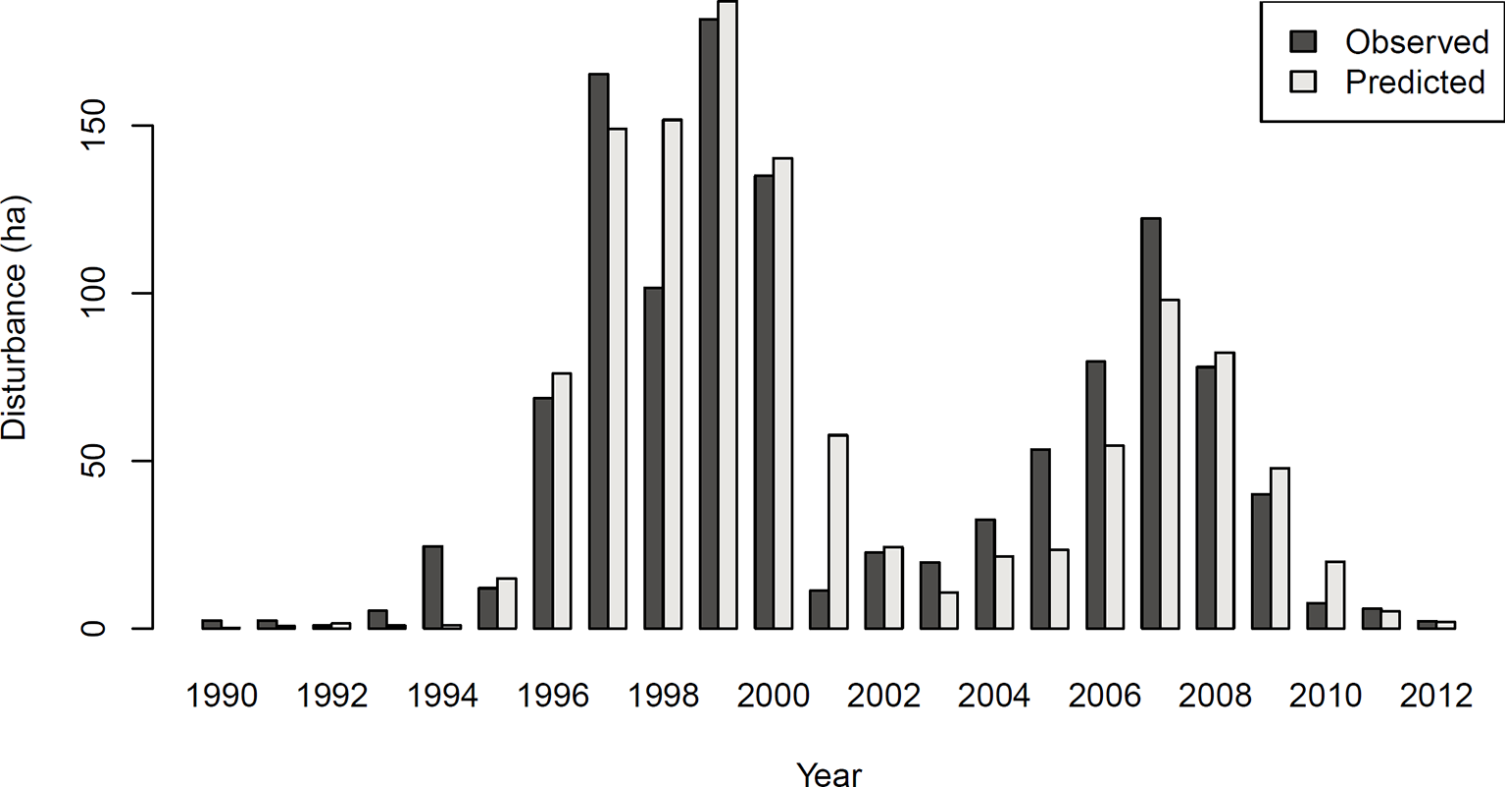
Observed and predicted area disturbed by bark beetles in Experiment 2 (N = 373,817).

The achieved accuracy was generally lower in Experiment 2 compared to Experiment 1 (**Table 2**). **Figure 4** shows a comparison of predicted and observed area disturbed over time. While the general pattern of two distinct outbreak waves within the 23-year study period was reproduced well by the DNN, the network had difficulties predicting the initial year of outbreak (early 1990s) and consistently underestimated the area disturbed during the gradation phase of the second outbreak wave (2003–2007). To provide additional context for the assessment of model performance, **Figure S3** in the **Supplementary Material** shows a similar time series comparison for the statistical model developed by Seidl et al. (2016). The Gleichlaeufigkeit of the DNN was with 0.750, which was slightly higher than that of the statistical model (0.727).

### Comparison With Other Machine Learning Algorithms

The DNN (**Table 2**) performed better than the other tested alternative algorithms in five of the six cases; only the random forest algorithm outperformed the DNN for Experiment 2 (see **Supplementary Table S1** in the **Supplementary Material** for performance metrics). Generally, the ensemble methods (gradient boosting machine and distributed random forest) were highly proficient in extracting meaningful information from the data. Compared to the ensemble models, the generalized linear model, applying a single linear model (albeit with many predictors) had considerably lower predictive power.

## Discussion

Deep learning is a new and powerful machine learning approach to model complex data. It is an approach under active development by a growing research community and is increasingly applied in a wide variety of fields (LeCun et al., 2015; Angermueller et al., 2016). Yet, it remains rarely used in ecology to date, as underscored by our review of the literature (see **Supplementary Material S1**). Specifically, we did not find a single study employing deep learning published in ecological flagship journals such as Ecology, Journal of Ecology, Ecology Letters, BioScience, Ecological Applications, Journal of Applied Ecology, Diversity and Distributions, or Global Ecology and Biogeography. However, deep learning has the potential to become a powerful tool for ecologists (Reichstein et al., 2019), especially as the field moves towards a more quantitative and predictive approach (Clark et al., 2001; Evans et al., 2012).

Deep learning approaches are good at generalizing beyond test data (Goodfellow et al., 2016), an ability that is of high importance in the context of prediction for applied ecological problems. A particular strength is the ability to achieve a high level of abstraction in raw data: Zhang et al. (2016), for instance, in a recent review in the context of remote sensing, concluded that DNNs are especially successful in high-level tasks such as object recognition, which are very difficult or even impossible to achieve with classic remote sensing approaches. Furthermore, deep learning may also contribute to more traditional approaches of ecological modelling for prediction, i.e. simulation modeling. For instance, DNNs could be used as powerful multi-dimensional “interpolators” to systematically analyze the growing number of simulation model ensembles (e.g., Warszawski et al., 2014), or build highly efficient meta-models of complex and computationally expensive existing simulation models (Marçais and de Dreuzy, 2017; Rammer and Seidl, 2019). Another promising approach is the hybridization of deep learning with process based models (Reichstein et al., 2019), e.g., by integrating deep learning-based sub models into a process based modeling framework, in order to advance ecological understanding.

A frequently stated reservation about applying ML in general and DNNs in particular relates to their black box character — the trained model and its weights cannot be interpreted in an intuitive way. Consequently, more traditional data models will remain an important means of inference, particularly for improving our understanding of relationships between drivers and responses in nature. It has to be noted, however, that such classical approaches make a priori assumptions on the underlying data model, which does not necessarily reflect the true relationships between driver variables and the response variable (Breiman, 2001). While conventional data models lead to more interpretable results and offer a more stringent framework for hypothesis testing, they are often characterized by a less accurate representation of reality. ML deduces relationships without making a priori assumptions about them, and is often better able to accurately describe the relationship between drivers and responses (Goodfellow et al., 2016). DNNs work particularly well with ecological data because of their ability to efficiently combine different types of data (e.g., image-like pixel data, numeric and categorical variables). In addition, their hierarchical multi-layer structure reflects the fact that ecosystems are frequently governed by a variety of processes along a nested hierarchy of scales (Raffa et al., 2008; Allen et al., 2014). We here found that DNNs outperformed all other approaches to modeling bark beetle outbreak dynamics (with the exception of another powerful ML algorithm, namely random forest).

Bark beetle outbreaks have increased considerably in many parts of the globe, and are expected to increase further under climate change (Seidl et al., 2017). Consequently, bark beetle outbreaks are a key concern for forest managers aiming to continuously supply ecosystem services to society (Hlásny et al., 2019). Precise and timely information about the probability of new bark beetle infestations would be a key asset for managing outbreaks, as it would allow a targeted application of containment measures such as sanitation logging and the deployment of pheromone traps. Here we show that DNNs have high predictive potential in the context of applied ecological issues such as bark beetle outbreaks. It has to be noted that we here deliberately excluded weather data from the vector of predictors, as such data are usually not readily available for managers. We rather focused on variables that are easily quantifiable in the field, such as infestations in the last two years, showing that already a small number of (spatially explicit) predictors can result in high predictive power when using DNNs. Future work could combine this approach with near-real time bark beetle risk mapping based on phenological models (e.g., Baier et al., 2007; Matthews et al., 2018) in order to account for weather-driven bark beetle development trajectories. Furthermore, the growing availability of remotely sensed information on past insect disturbances (Senf et al., 2017) could be integrated into future predictions of bark beetle dynamics. DNNs provide an ideal platform for such a comprehensive bark beetle forecasting system, as they are well suited to synthesize information from a wide variety of data sources (Goodfellow et al., 2016).

Deep learning is a rapidly evolving field, and DNNs have high potential beyond their application in predictive ecology. DNNs are, for instance, promising approaches for unsupervised learning (Sutskever et al., 2015), i.e. finding previously not identified patterns in data. This is particularly important in the context of the advent of big data in ecology, as the majority of big data is unlabeled. Consequently, unsupervised learning approaches in which DNNs are used to find patterns in data are expected to gain importance in the future (LeCun et al., 2015). More generally, it has been suggested that ML approaches can be applied in all stages of the scientific process, from observation to hypothesis creation and testing, to model building and prediction (Mjolsness et al., 2001). A deeper integration of ML in ecology could thus result in advances that go considerably beyond improved predictions (Peters et al., 2014). An increasing adoption of ML – and especially deep learning – can be found throughout science, technology and commerce, fueling the rapid development of methods, software, and even specialized hardware (Jordan and Mitchell, 2015). Given the substantial capacity of deep learning for ecological prediction, we maintain that adding deep learning to our arsenal of ecological methods could provide a substantial boost for quantitative ecology.

## Author Contributions

WR and RS jointly designed and wrote the paper. WR performed the neural network analysis.

## Conflict of Interest

The authors declare that the research was conducted in the absence of any commercial or financial relationships that could be construed as a potential conflict of interest.

## References

[B1] Abadi M. Agarwal A. Barham P. Brevdo E. Chen Z. Citro C. (2016). TensorFlow: large-scale machine learning on heterogeneous distributed systems. 1, 19.

[B2] Allen C. R. Angeler D. G. Garmestani A. S. Gunderson L. H. Holling C. S. (2014). Panarchy: theory and application. Ecosystems 17, 578–589. 10.1007/s10021-013-9744-2

[B3] Allouche O. Tsoar A. Kadmon R. (2006). Assessing the accuracy of species distribution models: prevalence, kappa and the true skill statistic (TSS). J. Appl. Ecol. 43, 1223–1232. 10.1111/j.1365-2664.2006.01214.x

[B4] Angermueller C. Pärnamaa T. Parts L. Stegle O. Oliver S. (2016). Deep learning for computational biology. Mol. Syst. Biol. 12, 878. 10.15252/msb.20156651 27474269 PMC4965871

[B5] Baier P. Pennerstorfer J. Schopf A. (2007). PHENIPS—A comprehensive phenology model of Ips typographus (L.) (Col., Scolytinae) as a tool for hazard rating of bark beetle infestation. For. Ecol. Manage. 249, 171–186. 10.1016/j.foreco.2007.05.020

[B6] Breiman L. (2001). Statistical modeling: the two cultures. Stat. Sci. 16, 199–231. 10.1214/ss/1009213726

[B7] Buras A. Wilmking M. (2015). Correcting the calculation of Gleichläufigkeit. Dendrochronologia 34, 29–30. 10.1016/j.dendro.2015.03.003

[B8] Clark J. S. Carpenter S. R. Barber M. Collins S. Dobson A. Foley J. A. (2001). Ecological forecasts: an emerging imperative. Science (80-. ). 10.1126/science.293.5530.657 11474103

[B9] Dietze M. C. Fox A. Beck-Johnson L. M. Betancourt J. L. Hooten M. B. Jarnevich C. S. (2018). Iterative near-term ecological forecasting: needs, opportunities, and challenges. Proc. Natl. Acad. Sci. 115, 1424–1432. 10.1073/pnas.1710231115 29382745 PMC5816139

[B10] Ershadi A. McCabe M. F. Evans J. P. Chaney N. W. Wood E. F. (2014). Multi-site evaluation of terrestrial evaporation models using FLUXNET data. Agric. For. Meteorol. 187, 46–61. 10.1016/j.agrformet.2013.11.008

[B11] Evans M. R. (2012). Modelling ecological systems in a changing world. Philos. Trans. R. Soc. B Biol. Sci. 367, 181–190. 10.1098/rstb.2011.0172 PMC3223798 22144381

[B12] Evans M. R. Norris K. J. Benton T. G. (2012). Predictive ecology: systems approaches. Philos. Trans. R. Soc. Lond. B. Biol. Sci. 367, 163–169. 10.1098/rstb.2011.0191 22144379 PMC3223810

[B13] Goodfellow I. Bengio Y. Courville A., (2016). Deep Learning. MIT Press.

[B14] H2O.ai, H2O: Scalable Machine Learning, 2017.

[B15] Hlásny T. Krokene P. Liebhold A. Montagné-huck C. Müller J. Qin H. (2019). Living with bark beetles: impacts, outlook and management options, From Science to Policy. European Forest Institute.

[B16] Ioffe S. Szegedy C. (2015). Batch Normalization: accelerating deep network training by reducing internal covariate shift. arXiv1502.03167, 1–11. 10.1007/s13398-014-0173-7.2

[B17] Johnson M. Schuster M. Le Q. V. Krikun M. Wu Y. Chen Z. (2016). Google’s Multilingual Neural Machine Translation System: Enabling Zero-Shot Translation 1–16.

[B18] Jordan M. I. Mitchell T. M. (2015). Machine learning: trends, perspectives, and prospects. Science (80-. ). 10.1126/science.aaa8415 26185243

[B19] Jordan R. Crall A. Gray S. Phillips T. Mellor D. (2015). Citizen science as a distinct field of inquiry. Bioscience. 10.1093/biosci/biu217

[B20] Kautz M. Dworschak K. Gruppe A. Schopf R. (2011). Quantifying spatio-temporal dispersion of bark beetle infestations in epidemic and non-epidemic conditions. For. Ecol. Manage. 262, 598–608. 10.1016/j.foreco.2011.04.023

[B21] Kennedy R. E. Andréfouët S. Cohen W. B. Gómez C. Griffiths P. Hais M. (2014). Bringing an ecological view of change to Landsat-based remote sensing. Front. Ecol. Environ. 12, 339–346. 10.1890/130066

[B22] Krizhevsky A. Sutskever I. Hinton G. E. (2012). ImageNet Classification with Deep Convolutional Neural Networks. Adv. Neural Inf. Process. Syst., 1–9. 10.1016/j.protcy.2014.09.007

[B23] LeCun Y. Bengio Y. Hinton G. (2015). Deep learning. Nature 521, 436–444. 10.1038/nature14539 26017442

[B24] Luo Y. Ogle K. Tucker C. Fei S. Gao C. LaDeau S. (2011). Ecological forecasting and data assimilation in a data-rich era. Ecol. Appl. 21, 1429–1442. 10.1890/09-1275.1 21830693

[B25] Marçais J. de Dreuzy J.-R. (2017). Prospective Interest of Deep Learning for Hydrological Inference. Groundwater 55, 688–692. 10.1111/gwat.12557 28732108

[B26] Matthews B. Netherer S. Katzensteiner K. Pennerstorfer J. Blackwell E. Henschke P. (2018). Transpiration deficits increase host susceptibility to bark beetle attack: experimental observations and practical outcomes for Ips typographus hazard assessment. Agric. For. Meteorol. 263, 69–89. 10.1016/j.agrformet.2018.08.004

[B27] Mjolsness E. DeCoste D. Mjolsness E. DeCoste D. Mjolsness E. DeCoste D. (2001). Machine Learning for Science: state of the art and future prospects. Science> (80-). 10.1126/science.293.5537.2051 11557883

[B28] Mouquet N. Lagadeuc Y. Devictor V. Doyen L. Duputié A. Eveillard D. (2015). Predictive ecology in a changing world. J. Appl. Ecol. 52, 1293–1310. 10.1111/1365-2664.12482

[B29] Nielsen M. A. (2015). Neural Networks and Deep Learning. Determination Press.

[B30] Olden J. D. Lawler J. J. Poff N. L. (2008). Machine learning methods without tears: a primer for ecologists. Q. Rev. Biol. 83, 171–193. 10.1086/587826 18605534

[B31] Peters D. P. C. Havstad K. M. Cushing J. Tweedie C. Fuentes O. Villanueva-Rosales N. (2014). Harnessing the power of big data: infusing the scientific method with machine learning to transform ecology. Ecosphere 5, 1–15. 10.1890/ES13-00359.1

[B32] Powers D. M. W. (2011). Evaluation: from precision, recall and F-measure to ROC, informedness, markedness & correlation. J. Mach. Learn. Technol. 2, 37–63. 10.9735/2229-3981

[B33] Raffa K. F. Aukema B. H. Bentz B. J. Carroll A. L. Hicke J. A. Turner M. G. (2008). Cross-scale Drivers of Natural Disturbances Prone to Anthropogenic Amplification: the dynamics of bark beetle eruptions. Bioscience 58, 501–517. 10.1641/B580607

[B34] Rammer W. Seidl R. (2019). A scalable model of vegetation transitions using deep neural networks. Methods Ecol. Evol. 2019, 1–12. 10.1111/2041-210X.13171 PMC6582592 31244986

[B35] Real L. E. Brown J. H., (1991). Foundations of ecology: classic papers with commentaries. Ecological Society of America, Chicago: University of Chicago Press.

[B36] Reichstein M. Camps-Valls G. Stevens B. Jung M. Denzler J. Carvalhais N. (2019). Deep learning and process understanding for data-driven Earth system science. Nature 566, 195–204. 10.1038/s41586-019-0912-1 30760912

[B37] Rumelhart D. E. Hinton G. E. Williams R. J. (1986). Learning representations by back-propagating errors. Nature 323, 533–536. 10.1038/323533a0

[B38] Seidl R. Müller J. Hothorn T. Bässler C. Heurich M. Kautz M. (2016). Small beetle, large-scale drivers: how regional and landscape factors affect outbreaks of the European spruce bark beetle. J. Appl. Ecol. 53, 530–540. 10.1111/1365-2664.12540 PMC4816203 27041769

[B39] Seidl R. Müller J. Hothorn T. Bässler C. Heurich M. Kautz M. (2015). Data from: small beetle, large-scale drivers: how regional and landscape factors affect outbreaks of the European spruce bark beetle. J. Appl. Ecol. 10.5061/dryad.c5g9s PMC4816203 27041769

[B40] Seidl R. Thom D. Kautz M. Martin-benito D. Peltoniemi M. Vacchiano G. (2017). Forest disturbances under climate change. Nat. Publ. Gr. 7, 395–402. 10.1038/nclimate3303 PMC5572641 28861124

[B41] Senf C. Seidl R. Hostert P. (2017). Remote sensing of forest insect disturbances: current state and future directions. Int. J. Appl. Earth Obs. Geoinf. 60, 49–60. 10.1016/j.jag.2017.04.004 28860949 PMC5572637

[B42] Shea K. Chesson P. (2002). Community ecology theory as a framework for biological invasions. Trends Ecol. Evol. 17, 170–176. 10.1016/s0169-5347(02)02495-3

[B43] Srivastava N. Hinton G. Krizhevsky A. Sutskever I. Salakhutdinov R. (2014). Dropout: a simple way to prevent neural networks from overfitting. J Mach. Learn Res. 15, 1929–1958. 10.1214/12-AOS1000

[B44] Steffen W. Richardson K. Rockstrom J. Cornell S. E. Fetzer I. Bennett E. M. (2015). Planetary boundaries: guiding human development on a changing planet. Science (80-). 10.1126/science.1259855 25592418

[B45] Sutskever I. Jozefowicz R. Gregor K. Rezende D. Lillicrap T. Vinyals O. (2015). Towards Principled Unsupervised Learning. arXiv 1–9.

[B46] Szegedy C. Ioffe S. Vanhoucke V. Alemi A. (2016). Inception-v4, Inception-ResNet and the Impact of Residual Connections on Learning. Arxiv 12.

[B47] Thom D. Seidl R. (2016). Natural disturbance impacts on ecosystem services and biodiversity in temperate and boreal forests. Biol. Rev. 91, 760–781. 10.1111/brv.12193 26010526 PMC4898621

[B48] Warszawski L. Frieler K. Huber V. Piontek F. Serdeczny O. Schewe J. (2014). The Inter-Sectoral Impact Model Intercomparison Project (ISI–MIP): project framework. Proc. Natl. Acad. Sci. 111, 3228–3232. 10.1073/pnas.1312330110 24344316 PMC3948262

[B49] Whitlock M. C. (2011). Data archiving in ecology and evolution: best practices. Trends Ecol. Evol. 26, 61–65. 10.1016/j.tree.2010.11.006 21159406

[B50] Williams J. W. Jackson S. T. (2007). Novel climates, no-analog communities, and ecological surprises. Front. Ecol. Environ. 5, 475–482. 10.1890/070037

[B51] Zhang L. Xia G.-S. Wu T. Lin L. Tai X. C. (2016). Deep Learning for Remote Sensing Image Understanding. J. Sensors 2016, 1–2. 10.1155/2016/7954154

