# Supplementary material for: Harnessing Deep Learning in Ecology: An Example Predicting Bark Beetle Outbreaks
Source: Front Plant Sci. 2019 Oct 28;10:1327. doi: 10.3389/fpls.2019.01327 (PMC6827389; doi:10.3389/fpls.2019.01327)
Supplement: Supplementary file 1 [file DataSheet_1.pdf]

Supplementary Material

Harnessing deep learning in ecology: An example predicting bark beetle outbreaks

Werner Rammer, Rupert Seidl

S1 - Deep learning applications in ecology

In order to evaluate the current usage of this method we searched the Scopus database of the subject areas environmental science, agricultural and biological sciences, as well as earth and planetary sciences for the keywords "deep learning", "deep neural networks" or "dnn". We filtered out reviews and papers that do not apply deep learning techniques, and grouped the remaining papers according to their main aim into "classification" and "prediction".

Deep learning approaches are increasingly used in ecology. The literature search yielded 151 candidate papers, of which 46 remained that actually apply deep learning techniques. Since the first deep learning applications in ecology published in 2016, the number of papers increased rapidly over the last three years (Figure S1). However, given the total number of 46 papers, the application of deep learning in ecology is still fairly limited. A majority of the reviewed papers (57%) used deep learning for classification. Classification tasks relied mostly on convolutional neural networks (CNNs) to classify images of plants or animals (88% of all classification tasks). When deep learning approaches were used for prediction (37% of papers), feedforward networks were typically applied. The objectives for which deep learning was used include the prediction of chemical properties of either air or water (e.g., ozone levels), or soil properties such as the height of the water table (53% of prediction tasks).

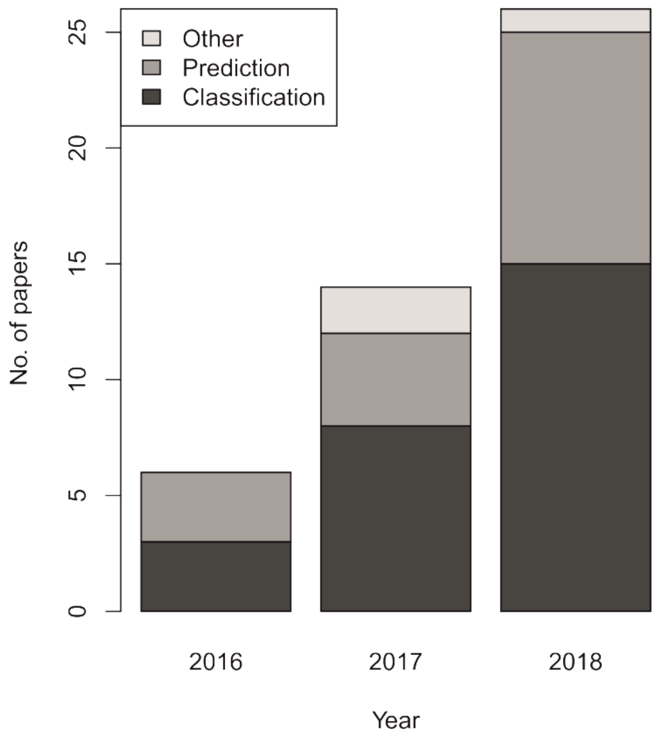

Figure S1. Number of papers found in Scopus using the search terms “deep learning”, “deep neural networks” or “dnn” in the subject areas environmental science, agricultural and biological sciences, as well as earth and planetary sciences. The category “Other” refers to papers that either mix prediction and classification, or apply DNNs for entirely different tasks (Date of search: 8.11.2018).

S2 – Deep learning resources for ecologists

DNN Training

The training of a DNN is an autonomous iterative process: First, in a forward pass, the network calculates an output $y^{(i)}$ (e.g., the label of an image) for every input vector $x^{(i)}$ (e.g., the raw pixels of the image). The input layer is set to $x^{(i)}$, and the neurons of each subsequent layer (2 to k) transform the output of the previous layer, producing an increasingly abstract representation of the data. Second, the deviation between expected and predicted values $\hat{y}_i$ -$y^{(i)}$ is calculated by applying a loss function (e.g., mean square error for regression tasks). The final step of an iteration is the backward pass, when the learning algorithm adjusts the individual weights w in order to minimize the difference between the networks' output $y^{(i)}$ and the expected output $\widehat{y^{(i)}}$. Mathematically, the back-propagation of the prediction error through the network is a practical application of the chain rule of differentiation. Algorithms such as stochastic gradient descent calculate a gradient vector for each weight w, indicating by what amount the prediction error would increase or decrease if the weight would change by a small amount. The algorithm then updates the weights by a fraction of the gradient (learning rate) in the direction of the steepest descent – averaged over several training examples – towards minimizing the error.

Over the course of many (typically tens of thousands to millions) iterations of learning the network will improve the representation of $y^{(i)} = f\left(x^{(i)}\right)$, as indicated by an increase in the training set accuracy. The ability to generalize, i.e., the networks' performance when confronted with examples of $x^{(j)}$ not contained in the training data, is periodically tested against an independent test dataset. Typically, the test set accuracy improves with training set accuracy, but eventually stops increasing or even starts to decrease again, which indicates overfitting of the training data. For prediction, usually the network with the best test set accuracy is used.

Designing and training of deep neural networks

In addition to the fundamental network type a number of additional parameters – referred to as hyper-parameters – need to be specified for training and predicting with DNNs (Bengio 2012). Generally, these parameters are problem-specific, although a range of default values exists (Angermueller *et al.* 2016). They are often set iteratively based on the performance of the DNN against the test dataset. The basic network architecture is determined by the number of layers and the number of neurons per layer. These should be chosen large enough for the model to learn the relationships in the training data while not overfitting the data. Typical ranges in current applications are five to 20 layers with 50 to 1000 neurons per layer. Furthermore, the user selects the activation function (i.e., the function that calculates the output of individual neurons) and the loss function (measuring the deviation between predicted and observed values in the learning process). Historically, the sigmoid function $(1/(1+\exp(-x))$ was frequently used as activation function. More recently, the rectified linear unit (ReLU, $f(x) = \max(x, 0)$) gained popularity due to improved performance in deep networks (LeCun, Bengio & Hinton 2015). The choice of the loss function is again problem-specific: A quadratic function is, for instance, well suited for regression tasks, while other functions such as the cross-entropy function (measuring the difference between the predicted and observed probability distributions) work well for classification.

The user can further adapt the network architecture by applying regularization strategies. Regularization aims at improving the network performance on the test dataset (i.e., its generality), possibly at the expense of decreasing training set performance (Goodfellow, Bengio & Courville 2016). One such technique is weight decay, which pushes the learned weights towards smaller values and thus facilitates simpler models (Nielsen 2015). Another important regularization technique is dropout (Srivastava *et al.* 2014). Here different subsets of the network are randomly deactivated during training, while the entire network is used for prediction. Dropout thus roughly

approximates the bagging approach often used in tree-based ML algorithms (Breiman 2001), and the final result can be thought of as an average over different sub-networks (Goodfellow, Bengio & Courville 2016). Furthermore, the training of the network can be influenced by choosing different optimization algorithms, and by prescribing the number of training iterations (also called epochs, that is the number of times the full training dataset is traversed), the batch size (i.e., the size of the subsets of the data simultaneously used for training), and the learning rate (i.e., how fast the weights can change during training) (Bengio 2012).

Practical considerations for deep learning applications

How well a DNN performs depends on the selection of an appropriate network architecture and sensible hyper-parameter values. Therefore, finding a good set-up for a given prediction problem is arguably the most challenging and time consuming part of applying deep learning approaches (Nielsen 2015). However, the increasing availability of software tools simplifies this iterative procedure, and a growing number of readily applicable defaults and guidelines provide good starting points for tailoring DNNs to different ecological questions. Some of those practical aspects are discussed below.

A growing number of open source software frameworks for the training and application of DNNs are available (e.g., *TensorFlow*, *Caffe*, *Theano*, *Torch7*, see Angermueller *et al.,* 2016 for an overview). Such frameworks typically provide the elements for building a network (such as "fully connected layers"), a variety of algorithms (optimizers, activation and loss functions, regularization layers, etc.), a number of examples (pre-defined networks with carefully selected hyper-parameters), and the means to execute training and prediction. All these elements can be easily combined using a scripting interface, often using the programming language Python. The training of deep neural networks is computationally expensive and can take from hours to weeks. Massive performance gains can be achieved by using generally

programmable graphics processing units (GPU), which are especially suited for the large matrix operations that form the mathematical core of deep learning. Modern deep learning frameworks allow a seamless integration of GPUs into the processing chain.

In addition to the availability of soft- and hardware for deep learning, the rapidly growing deep learning community increasingly provides helpful resources for DNN applications. This includes manuals and best practice examples for improving training performance and increasing the generalization power, and tools for analyzing the internal states of DNNs (i.e., shedding light into the "black box"). Deep learning is currently evolving at a very high pace (LeCun, Bengio & Hinton 2015), not least because of a strong commitment to an open source philosophy in the field. The growing variety of approaches and DNN-specific jargon might be an initial obstacle for ecologists, but excellent resources such as the works by Nielsen (2015), Angermueller *et al.* (2016), and Goodfellow et al. (2016) provide accessible starting points for obtaining a deeper understanding of the approach.

Machine learning libraries

We used the open source machine learning library TensorFlow (https://www.tensorflow.org/ (Abadi *et al.* 2016) developed by Google Inc. TensorFlow provides a Python API to declare the computational graph specifying the network structure by combining individual pre-defined components (e.g., convolutional or fully connected layers, loss-functions, regularization layers, etc.). The thus defined graph is then executed on either CPU, GPU, or even on different machines in parallel. To further simplify the use of TensorFlow, specialized higher level libraries have been developed. When such a higher level library is used, the process of setting up and training DNNs is reduced to providing a few lines of (typically) Python code. In this example we used the TFLearn library (http://tflearn.org/). Our example code can be found at https://github.com/werner-rammer/BBPredNet.

S3 Additional results of predicting bark beetle outbreaks

Predicting bark beetle disturbance from infestation maps (see Figure S3 in the main paper) can be viewed as a specific case of an image classification problem, where the network is asked to classify the focal cell of an example image either as disturbed or undisturbed. Neural Networks using convolutional layers (Convolutional Neural Networks, CNNs) have been applied successfully for image classification recently (e.g., Krizhevsky, Sutskever & Hinton 2012; Szegedy et al. 2016), and were thus selected as the network type here. We used the dataset of Experiment 1 (setting aside individual years) for evaluating different network architectures. The hyper-parameters evaluated iteratively were network capacity (number of layers and neurons per layer), applied regularization techniques, as well as the used loss function and optimizer. The training of the individual candidate networks was stopped when the accuracy of the network on the test dataset did not increase further. The thus determined network architecture was also used for Experiment 2. All experiments and predictions were conducted using the TensorFlow framework and run on a desktop PC with an Intel QuadCore CPU (Intel i5-6600) and equipped with a NVidia GTX 1070 GPU.

The final network architecture chosen was a convolutional neural network with five convolution layers, followed by five fully connected layers and a final softmax layer (Nielsen, 2015) for classification. We used categorical cross entropy as the cost function, and weight decay (Nielsen, 2015), dropout (Srivastava et al., 2014), and batch normalization (Ioffe and Szegedy, 2015) to improve generalization. Figure S2 shows the schematic structure of the DNN architecture. The presented network efficiently combines image-like pixel data with additional variables that are both numerical (climate variables) or categorical (outbreak stages). We trained the final architecture for 60 epochs, which took approximately one hour on the hardware used, and selected the

epoch with the highest test set accuracy for prediction. The GitHub repository (https://github.com/werner-rammer/BBPredNet) contains the full source code for reproducing this example, and includes further details on data preprocessing and the final network architecture.

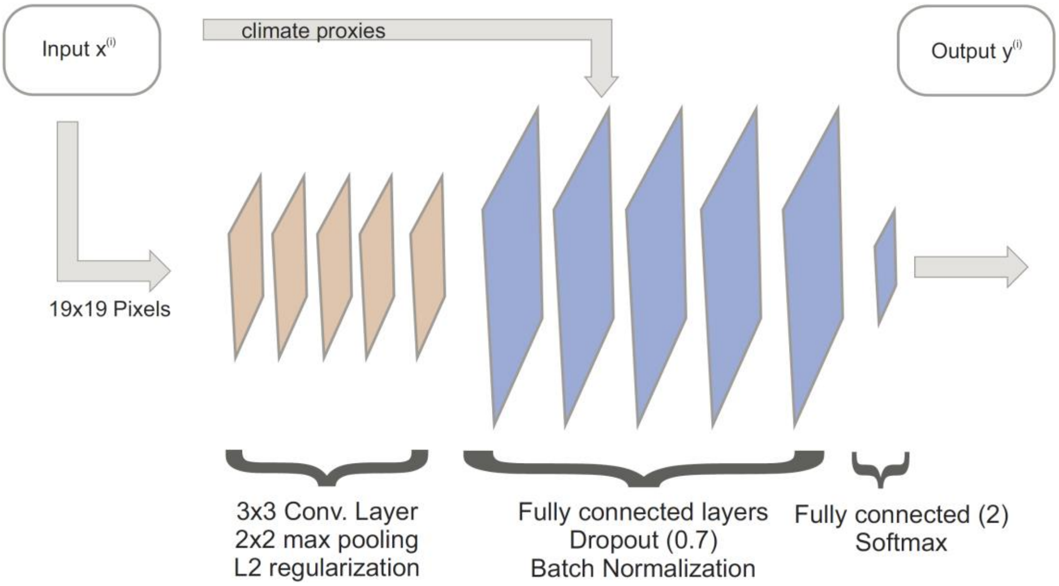

Figure S2. Schematic structure of the applied Deep Learning Network. For more details we refer to the GitHub repository.

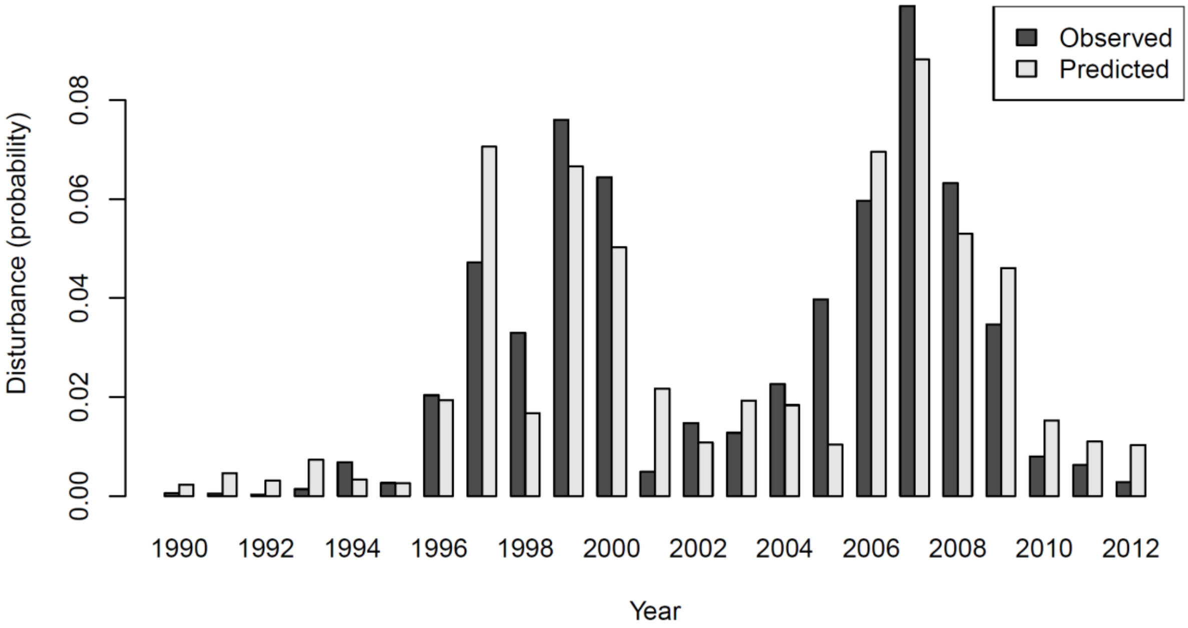

Figure S3: Observed and predicted disturbance probability from statistical modelling as published by Seidl et al. (2016). Compare with Figure 4 showing DNN based predictions in the main paper.

Table S1. Performance comparison of DNN with alternative machine learning methods. DNN=deep neural network (this contribution), DRM=distributed random forest, GBM=gradient boosting machine, GLM=generalized linear model.

Algorithm	Parameter	Experiment 1 (n= 292,559)	Experiment 2 (n=373,817)
DNN	Accuracy	0.966	0.959
	Precision	0.652	0.413
	Recall	0.392	0.411
	F1 Score	0.490	0.412
GBM	Accuracy	0.949	0.951
	Precision	0.307	0.339
	Recall	0.427	0.417
	F1 Score	0.357	0.374
DRM	Accuracy	0.943	0.976
	Precision	0.267	0.712
	Recall	0.408	0.569
	F1 Score	0.323	0.569
GLM	Accuracy	0.928	0.940
	Precision	0.234	0.203
	Recall	0.324	0.250
	F1 Score	0.272	0.224

References

Abadi, M., Barham, P., Chen, J., Chen, Z., Davis, A., Dean, J., Devin, M., Ghemawat, S., Irving, G., Isard, M., Kudlur, M., Levenberg, J., Monga, R., Moore, S., Murray, D.G., Steiner, B., Tucker, P., Vasudevan, V., Warden, P., Wicke, M., Yu, Y. & Zheng, X. (2016). TensorFlow: A system for large-scale machine learning. *Osdi '16 Proceedings*.

Angermueller, C., Pärnamaa, T., Parts, L., Stegle, O. & Oliver, S. (2016). Deep learning for computational biology. *Molecular Systems Biology*, **12**, 878.

Bengio, Y. (2012). Practical recommendations for gradient-based training of deep architectures. *Lecture Notes in Computer Science*, 437–478.

Breiman, L. (2001). Statistical modeling: The two cultures. *Statistical Science*, **16**, 199–231.

Goodfellow, I., Bengio, Y. & Courville, A. (2016). *Deep Learning*. MIT Press.

LeCun, Y., Bengio, Y. & Hinton, G. (2015). Deep learning. *Nature*, **521**, 436–444.

Nielsen, M.A. (2015). *Neural Networks and Deep Learning*. Determination Press.

Seidl, R., Müller, J., Hothorn, T., Bässler, C., Heurich, M. & Kautz, M. (2016). Small beetle, large-scale drivers: How regional and landscape factors affect outbreaks of the European spruce bark beetle. *Journal of Applied Ecology*, **53**, 530–540.

Srivastava, N., Hinton, G., Krizhevsky, A., Sutskever, I. & Salakhutdinov, R. (2014). Dropout: A Simple Way to Prevent Neural Networks from Overfitting. *Journal of Machine Learning Research*, **15**, 1929–1958.
